# Profiling membrane glycerolipids during γ-ray-induced membrane injury

**DOI:** 10.1186/s12870-017-1153-9

**Published:** 2017-11-15

**Authors:** Guowei Zheng, Weiqi Li

**Affiliations:** 1 Key Laboratory for Plant Diversity and Biogeography of East Asia, Kunming Institute of Botany, Chinese Academy of Sciences, Kunming, Yunnan 650201 People’s Republic of China; 2 Germplasm Bank of Wild Species, Kunming Institute of Botany, Chinese Academy of Sciences, Kunming, Yunnan 650201 People’s Republic of China

**Keywords:** Gamma irradiation, Membrane injury, Phospholipase D, Lipidomics, Plastidic lipids, Extraplastidic lipids

## Abstract

**Background:**

γ-rays are high-energy radiation that cause a range of random injuries to plant cells. Most studies on this issue have focused on γ-ray-induced nucleotide damage and the production of reactive oxygen species in cells, so little is known about the glycerolipid metabolism during γ-rays induced membrane injury. Using an ESI-MS/MS-based lipidomic method, we analysed the lipidome changes in wild-type and phospholipase D (PLD)δ- and α1-deficient *Arabidopsis* after γ-ray treatment. The aim of this study was to investigate the role of PLD-mediated glycerolipid metabolism in γ-ray-induced membrane injury.

**Results:**

The ion leakage of *Arabidopsis* leaves after 2885-Gy γ-ray treatment was less than 10%. High does γ-ray treatment could induce the accumulation of intracellular reactive oxygen species (ROS). Inhibition of PLDα1 caused severe lipid degradation under γ-ray treatment. γ-ray-induced glycerolipid degradation mostly happened in chloroplastidic lipids, rather than extraplastidic ones. The levels of lysophosphatidylcholine (lysoPC) and lysophosphatidylethanolamine (lysoPE) were maintained in the WS ecotypes during γ-ray treatments, while increased significantly in the Col ecotype treated with 1100 Gy. After 210- and 1100-Gy γ-ray treatments, the level of lysophosphatidylglycerol (lysoPG) decreased significantly in the four genotypes of *Arabidopsis*.

**Conclusions:**

γ-ray-induced membrane injury may occur via an indirect mechanism. The degradation of distinct lipids is not synchronous, and that interconversions among lipids can occur. During γ-ray-induced membrane injury, the degradation of phosphatidylcholine (PC) and phosphatidylethanolamine (PE) may be mediated by PLDζ1 or phospholipase A1. The degradation of phosphatidylglycerol was not mediated by PLA, PLDδ or PLDα1, but by phospholipase C or other PLDs. γ-rays can decrease the double-bond index and increase the acyl chain length in membrane lipids, which may make membranes more rigid and further cause injury in membranes.

**Electronic supplementary material:**

The online version of this article (10.1186/s12870-017-1153-9) contains supplementary material, which is available to authorized users.

## Background

Gamma irradiation (γ-rays) from ^60^Co involves exposure to high-energy photons and is widely used to induce mutations in crops and flowers [1–3]. It is also extensively applied to extend the shelf life of food by altering its biochemical metabolism and destroying microorganisms [4–6]. The targeting of γ-rays to plant cells is completely non-specific and the response of the plant is random [7]. γ-rays can directly induce DNA damage and influence genome structure due to their high energy [8, 9]. They can also induce the production of free radicals and reactive oxygen species (ROS), which indirectly disturb the physiological and biochemical properties of cells and have deleterious effects on the plant [10–13].

Membranes are sensitive to environmental changes, and abiotic stresses directly affect membrane properties. Glycerolipids are the main constituents of membranes; adjustments of the composition, unsaturation and acyl chain length, enables plant to keep the integrity and fluidity of their membranes under environmental stresses [14–16]. During freezing treatment, plants tend to synthesise glycerolipids with a larger head group to maintain the integrity of their membranes [17]. In addition, the level of galactolipids and the degree of unsaturation were found to decline in the alpine plant *Meconopsis racemosa* after its introduction to a lowland region [18], and abscisic acid (ABA)-promoted leaf senescence in *Arabidopsis* was shown to be retarded by attenuating lipid degradation [19]. However, little is known about how membrane glycerolipids respond to γ-rays.

Phospholipase D (PLD) is one of the most important enzymes regulating phospholipid metabolism. The PLD family in plants comprises 12 members, which are classified into six types: PLDα (3), β (2), γ (3), δ, ε and ζ (2). PLD and its hydrolysate, phosphatidic acid (PA), play important roles in plant responses to drought, cold and salinity [20–24]. Different PLD isoforms might play different regulatory roles in plants [25]. PLDα1 and δ are the most abundant PLDs [26], and they play complex roles during plant responses to stress. An *Arabidopsis* mutant with suppressed PLDα1 was found to be tolerant to cold, while a PLDδ antisense mutant was sensitive to it [22, 27]. In addition, suppression of both PLDα1 and PLDδ was found to retard ABA- and ethylene-promoted senescence of detached leaves in *Arabidopsis* [19, 28]. It is reported that γ-rays could induce microsomal membranes deterioration in cauliflower florets [29]. However, little is known about the involvement of PLDα1 and PLDδ during γ-ray-induced membrane injury.

In the present study, we treated wild-type (Columbia and Wassilewskija) and PLD-deficient mutant (*PLDδ-def* and *PLDα1-def*) *Arabidopsis* with γ-rays at doses of 46, 210 and 1100 Gy. The two higher doses were previously reported to cause growth retardation and inhibition of bolting in *Arabidopsis*, respectively [30]. Using an lipidomic analysis based on electrospray tandem mass spectrometry (ESI-MS/MS) [31], we investigated the changes of membrane glycerolipid profiles after these different γ-ray treatments. The following two questions were addressed in this work: (i) How do membrane lipids respond to abiotic stresses of γ-rays? (ii) Do PLDs participate in plant responses to γ-rays?

## Results

### γ-ray-induced membrane injury may occur via an indirect mechanism

**Fig. 1:**
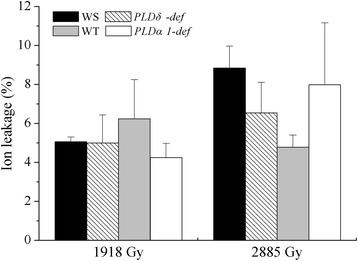
Ion leakage of *Arabidopsis* with different genotypes treated with different doses of γ-rays. WS, Wassilewskija ecotype; *PLDδ-def*, *PLDδ-knockout* mutant with WS background; Col, Columbia ecotype; *PLDα1-def*, *PLDα1-deficient* mutant with Col background

γ-rays are high-energy ion radiation that may damage membranes. Ion leakage monitored by the relative conductivity is an effective method to detect such damage. We thus investigated the ion leakage of *Arabidopsis* irradiated with γ-rays at 1918 and 2885 Gy, two higher doses that could inhibit growth in *Arabidopsis* (Additional file 1: Fig. S1). Surprisingly, the ion leakage of leaves treated with these two doses was less than 10%, and the differences in ion leakage between wild-type and mutant plants were not statistically significant (Fig. 1). Immediately after γ-rays treatment, the intracellular reactive oxygen species (ROS) increased significantly (Additional file 2: Fig. S2), which indicate that γ-rays caused serious oxidative stresses in *Arabidopsis*. However, the level of lipid peroxidation indicated by malondialdehyde (MDA) affected little by γ-rays treatment (Additional file 3: Fig. S3). It was suspected that peroxidation of lipid may not happen immediately after γ-rays treatment. These results indicate that γ-rays may not cause membrane leakage and that γ-ray-induced injury to *Arabidopsis* membranes may be indirect.

### Large changes in lipid profiles occurred after γ-ray treatments

**Fig. 2:**
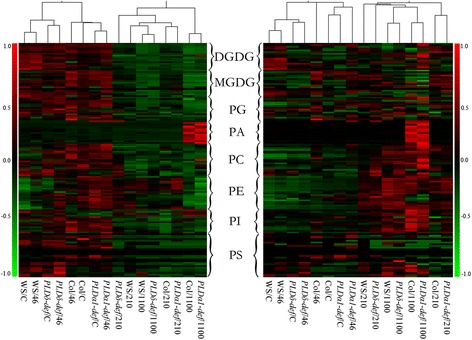
Profiles of glycerolipid levels in different *Arabidopsis* genotypes treated with different doses of γ-rays. *Left panel*: absolute levels of lipids (nmol per mg dry weight). *Right panel*: relative levels of lipids (mol%). Each column represents a specific *Arabidopsis* genotype after certain dose of γ-ray treatment, and each row represents a specific lipid molecular species. Data are normalized row-wise to the median value for different plants and treatments. The 139 lipid molecular species of various genotypes and γ-rays treatments were showed. WS, Wassilewskija ecotype; *PLDδ-def*, *PLDδ-knockout* mutant with WS background; Col, Columbia ecotype; *PLDα1-def*, *PLDα1-deficient* mutant with Col background. “C” represents control untreated plants. “46”, “210” and “1100” represent samples treated with γ-rays at 46, 210 and 1100 Gy

To investigate γ-rays induced membrane injury, three dose of γ-rays (46, 210 and 1100 Gy) were applied. Among which, 46 Gy γ-ray had little effect on the growth of *Arabidopsis*, while 210 Gy γ-ray caused growth retardation, and 1100 Gy γ-ray resulted in inhibition of bolting in *Arabidopsis* [30]. The metabolism of membrane lipids is an important way in which plants respond to environmental stresses. PLDδ and PLDα1 are two important enzymes that participate in membrane lipid metabolism. To understand better how PLDδ and PLDα1 function during γ-ray-induced membrane glycerolipid metabolism, we did a lipidome analysis of 11 classes that contain about 150 molecular species of membrane glycerolipids. Hierarchical clustering analysis (HCA) of the lipid profiles was used to obtain an overview of the effects of γ-irradiation treatment in conjunction with PLD deficiency (Fig. 2). Changes in both the absolute levels of these lipids (nmol/mg dry weight), which reflect lipid degradation, and their relative levels (mol%), which can reflect inter-conversion among lipids, were visualized using HCA (Fig. 2). The two ecotypes of *Arabidopsis* were separated in terms of both absolute and relative levels.

The data shown in Fig. 2 reveal that there were considerable changes in lipid molecular species during γ-ray-induced membrane injury, with most lipids being degraded (Fig. 2, left panel). However, in Col and PLDα1 mutants treated with γ-rays at 1100 Gy, the level of PA increased markedly. In the right-hand panel, which shows the relationships among the profiles of relative lipid levels (mol%) of different genotypes of *Arabidopsis* subjected to various γ-ray treatments, the profiles differ from those of the absolute levels. The levels of chloroplastidic lipids, such as monogalactosyldiacylglycerol (MGDG), digalactosyldiacylglycerol (DGDG) and some phosphatidylglycerol (PG) molecules, decreased. However, the level of most phospholipids increased, including PC, PE, phosphatidylinositol (PI), and some molecules of PG and phosphatidylserine (PS); these increases may have been caused by the degradation of galactolipids, which are the main glycerolipids in cells. These results may indicate that the chloroplastidic lipids were those most rapidly degraded during γ-ray-induced membrane injury.

### Lipid degradation during γ-ray-induced membrane injury

**Fig. 3:**
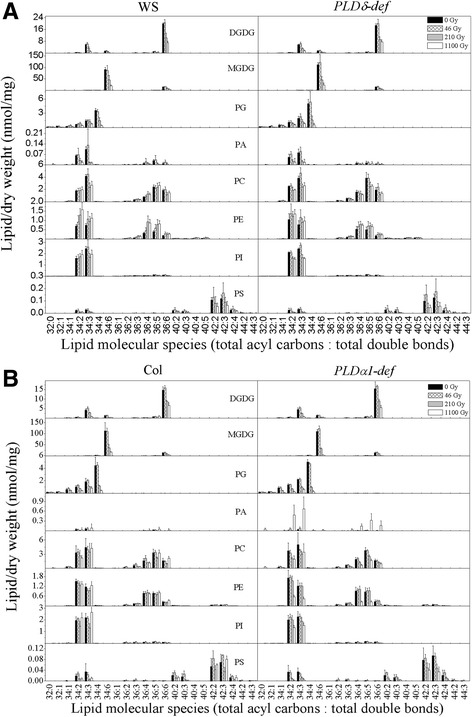
Changes in lipid molecular species after γ-ray treatments of WS and PLDδ-def (**a**) and Col and PLDα1-def (**b**). The dry weight is the dry weight of leaves after lipid extraction. Data are mean ± standard deviation (*n* = 4 or 5). WS, Wassilewskija ecotype; *PLDδ-def*, *PLDδ-knockout* mutant with WS background; Col, Columbia ecotype; *PLDα1-def*, *PLDα1-deficient* mutant with Col background

Detailed analysis of the glycerolipidome indicates that phospholipids and galactolipids showed similar changes in the two ecotypes between control and 46-Gy-treated plants (Fig. 3), except for some PE molecules, which increased in the Wassilewskija (WS) ecotype after 46-Gy γ-ray treatment (Fig. 3a; Table 1) but decreased or were unchanged in the Columbia ecotype (Col) (Fig. 3b; Table 1).

**Table 1:** Total content of each lipid class after γ-ray treatment at different doses in WS, *PLDδ-deficient* (def), Col and *PLDα1-def* plants

Lipid species	Genotype	Dose of gamma irradiation (Gy)				
		0	46	210	1100	RC (%)
		Lipid content (nmol per mg dry weight)				
DGDG	WS	29.90 ± 1.11^aA^	31.67 ± 3.23^aA^	18.08 ± 1.20^bA^	10.05 ± 1.52^cA^	−66.39
	*PLDδ-def*	28.61 ± 1.90^aA^	31.05 ± 4.47^aAB^	14.27 ± 2.94^bB^	11.18 ± 2.25^bA^	−60.92
	Col	22.87 ± 2.55^aB^	25.70 ± 2.53^aB^	13.86 ± 0.91^bB^	9.60 ± 1.65^cA^	−58.02
	*PLDα1-def*	24.20 ± 5.25^aB^	26.71 ± 1.60^aB^	13.71 ± 1.10^bB^	8.40 ± 1.94^cA^	−65.29
MGDG	WS	110.89 ± 7.72^aA^	109.24 ± 21.60^aB^	67.94 ± 5.03^bA^	27.38 ± 3.84^cA^	−75.31
	*PLDδ-def*	131.39 ± 8.28^aA^	127.81 ± 9.83^aAB^	57.18 ± 16.82^bAB^	30.00 ± 9.49^cA^	−77.17
	Col	132.29 ± 39.34^aA^	135.45 ± 13.70^aAB^	49.12 ± 14.42^bAB^	22.76 ± 2.31^bA^	−82.80
	*PLDα1-def*	131.57 ± 12.34^bA^	148.51 ± 14.34^aA^	44.73 ± 4.67^bB^	6.31 ± 1.54^cB^	−95.20
PG	WS	6.47 ± 0.68^aB^	6.39 ± 0.89^aC^	5.04 ± 0.32^bA^	2.18 ± 0.26^cA^	−66.31
	*PLDδ-def*	9.16 ± 1.59^aA^	8.48 ± 0.95^aB^	4.39 ± 1.55^bAB^	2.45 ± 0.66^cA^	−73.25
	Col	8.28 ± 2.10^aAB^	8.95 ± 0.12^aAB^	3.77 ± 0.04^bAB^	2.06 ± 0.35^cAB^	−75.12
	*PLDα1-def*	9.88 ± 0.54^aA^	9.76 ± 0.29^aA^	3.37 ± 0.42^bB^	1.48 ± 0.44^cB^	−85.02
PA	WS	0.21 ± 0.02^abA^	0.28 ± 0.21^aA^	0.06 ± 0.03^bcA^	0.04 ± 0.03^cA^	−80.95
	*PLDδ-def*	0.16 ± 0.06^aAB^	0.20 ± 0.05^aA^	0.05 ± 0.01^bAB^	0.05 ± 0.07^bA^	−68.75
	Col	0.15 ± 0.03^aB^	0.13 ± 0.02^aA^	0.03 ± 0.02^aAB^	0.24 ± 0.34^aA^	60.00
	*PLDα1-def*	0.07 ± 0.04^aC^	0.19 ± 0.08^aA^	0.03 ± 0.01^aB^	1.57 ± 1.31^aA^	2142.86
PC	WS	12.02 ± 1.01^aB^	12.34 ± 2.50^aB^	9.84 ± 2.67^aA^	9.85 ± 0.86^aB^	−18.05
	*PLDδ-def*	14.20 ± 0.78^aAB^	15.45 ± 0.78^aA^	9.36 ± 1.02^bA^	10.46 ± 1.13^bB^	−26.33
	Col	16.10 ± 4.20^aAB^	16.72 ± 0.52^aA^	10.12 ± 0.49^bA^	13.96 ± 3.02^abA^	−13.29
	*PLDα1-def*	17.84 ± 5.66^aA^	16.56 ± 0.59^abA^	10.51 ± 0.38^bcA^	9.77 ± 3.17^cB^	−45.24
PE	WS	2.58 ± 0.32^bB^	3.67 ± 1.32^abB^	4.69 ± 1.44^aA^	4.67 ± 0.40^aA^	81.01
	*PLDδ-def*	3.41 ± 0.97^aB^	4.67 ± 1.70^aAB^	4.79 ± 0.43^aA^	4.41 ± 0.69^aA^	29.33
	Col	5.03 ± 0.50^aA^	4.57 ± 0.52^aAB^	4.35 ± 0.85^aA^	4.95 ± 0.66^aA^	−1.59
	*PLDα1-def*	5.61 ± 1.60^aA^	5.87 ± 0.71^aA^	5.59 ± 1.00^aA^	1.83 ± 0.32^bB^	−67.38
PI	WS	4.44 ± 0.43^aC^	4.54 ± 1.10^aA^	3.99 ± 0.27^aAB^	4.17 ± 0.81^aB^	−6.08
	*PLDδ-def*	5.01 ± 0.12^bAB^	5.51 ± 0.05^aA^	3.58 ± 0.41^cAB^	3.33 ± 0.27^cBC^	−33.53
	Col	4.66 ± 0.63^aBC^	4.89 ± 0.20^aA^	3.33 ± 0.26^bB^	5.21 ± 0.91^aA^	11.80
	*PLDα1-def*	5.26 ± 0.27^aA^	5.20 ± 0.44^aA^	4.22 ± 0.72^bA^	3.09 ± 0.46^cC^	−41.25
PS	WS	0.37 ± 0.07^abA^	0.45 ± 0.23^aA^	0.25 ± 0.12^abA^	0.18 ± 0.09^bA^	−51.35
	*PLDδ-def*	0.38 ± 0.14^aA^	0.54 ± 0.27^aA^	0.16 ± 0.10^bAB^	0.16 ± 0.08^bA^	−57.89
	Col	0.25 ± 0.11^abA^	0.34 ± 0.01^aA^	0.11 ± 0.04^cB^	0.19 ± 0.03^bcA^	−24.00
	*PLDα1-def*	0.39 ± 0.11^aA^	0.36 ± 0.04^aA^	0.12 ± 0.02^bB^	0.09 ± 0.03^bA^	−76.92
Total	WS	167.31 ± 8.67^aA^	168.94 ± 28.87^aB^	111.77 ± 5.48^bA^	56.29 ± 3.57^cA^	−66.36
	*PLDδ-def*	193.47 ± 11.35^aA^	192.12 ± 11.80^aAB^	94.64 ± 24.19^bAB^	62.21 ± 12.26^cA^	−67.85
	Col	189.87 ± 47.98^aA^	197.15 ± 15.57^aAB^	86.00 ± 10.78^bB^	59.78 ± 10.33^bA^	−68.52
	*PLDα1-def*	194.49 ± 24.18^aA^	214.50 ± 15.16^aA^	79.35 ± 3.34^bB^	34.55 ± 5.80^cB^	−82.24

The RC is the percentage value of the 1100-Gy-treated plants compared with the control (0 Gy). Different lowercase letters marked in the same row represent significantly different among different γ-ray treatments. Different uppercase letters marked in the same column represent significantly different among different genotypes, (*p* < 0.05). Data are means ± standard deviation (*n* = 4 or 5)

For the two doses of γ-rays (210 and 1100 Gy) treatment, most lipids degraded during treatment in the two ecotypes. Inhibition of PLDα1 caused severe lipid degradation under γ-ray treatment. After treatment at 1100 Gy, the decrease of total lipids in the PLDα1 mutant was 82.24%, whereas in the other three genotypes, this decrease was about 68% (Table 1). The degradation of galactolipids was more severe than that of phospholipids, and the ratio of these two kinds of lipids decreased significantly (Table 2). For the two galactolipids, the degradation of MGDG occurred more rapidly than that of DGDG, which caused the significantly decrease of the ratio of MGDG to DGDG (Table 2). Chloroplastidic lipids were the most degraded, most of them decreasing by 60% (Table 1). 34:3 DGDG, 36:6 DGDG, 34:6 MGDG and 34:4 PG, the main chloroplastidic lipids, were the most degraded (Fig. 3).

**Table 2:** Lipid ratios after different doses of γ-ray treatment in WS, *PLDδ-deficient* (def), Col and *PLDα1-def* plants

Lipid species	Genotype	Dose of gamma irradiation (Gy)			
		0	46	210	1100
		Ratio			
Galactolipid/phospholipid	WS	5.41 ± 0.29^aA^	5.18 ± 0.81^aA^	3.14 ± 0.61^bA^	1.68 ± 0.18^cAB^
	*PLDδ-def*	4.73 ± 0.17^aB^	4.69 ± 0.47^aA^	3.09 ± 0.54^bA^	1.97 ± 0.51^cA^
	Col	4.64 ± 0.14^aB^	4.45 ± 0.48^aA^	2.88 ± 1.01^bA^	1.24 ± 0.13^cBC^
	*PLDα1-def*	4.12 ± 0.52^aC^	4.64 ± 0.54^aA^	2.41 ± 0.20^bA^	0.81 ± 0.10^cC^
MGDG/DGDG	WS	3.72 ± 0.39^aB^	3.42 ± 0.46^aC^	3.58 ± 0.52^aA^	2.74 ± 0.30^bA^
	*PLDδ-def*	4.61 ± 0.41^aAB^	4.58 ± 0.58^aB^	4.16 ± 0.08^aA^	2.65 ± 0.40^bA^
	Col	5.70 ± 1.25^aA^	5.27 ± 0.10^aAB^	3.57 ± 1.19^bA^	2.48 ± 0.26^bA^
	*PLDα1-def*	5.59 ± 0.91^aA^	5.55 ± 0.25^aA^	3.26 ± 0.14^bA^	0.77 ± 0.24^cB^
PC/PE	WS	4.68 ± 0.38^aA^	3.21 ± 0.26^bA^	2.13 ± 0.36^cA^	2.10 ± 0.18^cB^
	*PLDδ-def*	3.99 ± 0.83^aAB^	3.27 ± 1.04^abA^	2.42 ± 0.47^bA^	2.39 ± 0.20^bB^
	Col	3.23 ± 0.95^aB^	3.61 ± 0.39^aA^	2.81 ± 1.15^aA^	2.81 ± 0.35^aB^
	*PLDα1-def*	3.22 ± 0.61^bB^	2.84 ± 0.29^bcA^	2.01 ± 0.20^cA^	5.27 ± 1.01^aA^

Different lowercase letters marked in the same row represent significantly different among different γ-ray treatments. Different uppercase letters marked in the same column represent significantly different among different genotypes, (p < 0.05). Data are means ± standard deviation (n = 4 or 5)

After 210 and 1100 Gy γ-rays treatments, some classes of molecules, such as 34:2 PI, increased in wild-type plants but decreased in the two mutants (Fig. 3). PE increased in WS and the PLDδ mutant, and decreased in Col and the PLDα1 mutant (Table 1). PC levels decreased during γ-ray-induced membrane injury and the level in the *PLDα1-def* mutant decreased the most, by 45.24% (Table 1). Levels of PA, which can be induced by stresses and as products of the degradation of other glycerolipids, were slightly decreased in WS and the *PLDδ-def* mutant. However, PA levels increased in Col and the *PLDα1-def* mutant, and this increase was more than 20 times greater in the *PLDα1-def* mutant after treatment with γ-rays at 1100 Gy (Table 1). The levels of most lipids declined, although some increased, both in absolute and in relative terms. This could suggest that the degradation of distinct lipids is not synchronous, and that interconversions among lipids can occur.

### Changes of lysophospholipids after γ-ray treatments

**Fig. 4:**
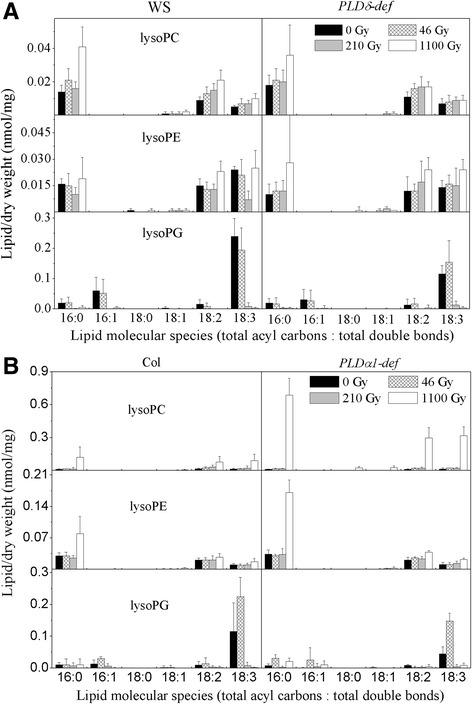
Changes in lysophospholipid molecular species after γ-ray treatments of WS ecotype (**a**) and Col ecotype (**b**). The dry weight is the dry mass of leaves after lipid extraction. Data are mean ± standard deviation (n = 4 or 5). WS, Wassilewskija ecotype; *PLDδ-def*, *PLDδ-knockout* mutant with WS background; Col, Columbia ecotype; *PLDα1-def*, *PLDα1-deficient* mutant with Col background

Lysophospholipids are minor phospholipids generated by the hydrolysis of phospholipids by phospholipase A1 (PLA1) [32], the level of which can increase several fold during exposure to stress [31]. The levels of lysoPC and lysoPE were maintained in the WS ecotypes (Fig. 4a; Table 3) during γ-ray treatments, while increased significantly in the Col ecotype treated with 1100 Gy (Fig. 4b; Table 3). The level of lysoPC increased by a factor of about 5 and 44, while the level of lysoPE increased by a factor of about 1.17 and 1.88, in Col and the *PLDα1-def* mutant, respectively (Table 3). The levels of 16:0, 18:2 and 18:3 lysoPC, and 16:0 lysoPE showed the greatest increases in the Col ecotype (Fig. 4b). The level of lysoPG, which hydrolyses PG by PLA1, remained unchanged in the WS ecotype after 46-Gy γ-ray treatment, but increased in the Col ecotype (Table 3). However, after 210- and 1100-Gy γ-ray treatments, the level of lysoPG decreased significantly in the four genotypes (Table 3). The level of 18:3 lysoPG showed the most marked decrease (Fig. 4b).

**Table 3:** Levels of lysophospholipids in leaves of *Arabidopsis* during γ-ray-induced senescence

Lipid species	Genotype	Dose of gamma irradiation (Gy)				
		0	46	210	1100	RC (%)
		nmol per mg dry weight				
LysoPC	WS	0.05 ± 0.01^abA^	0.04 ± 0.01^bB^	0.03 ± 0.01^bA^	0.07 ± 0.03^aB^	40.00
	*PLDδ-def*	0.04 ± 0.02^bA^	0.04 ± 0.01^bB^	0.03 ± 0.01^bA^	0.06 ± 0.01^aB^	50.00
	Col	0.05 ± 0.01^abA^	0.06 ± 0.01^aAB^	0.07 ± 0.05^aA^	0.30 ± 0.21^bB^	500.00
	*PLDα1-def*	0.03 ± 0.02^bA^	0.06 ± 0.01^bA^	0.06 ± 0.02^bA^	1.35 ± 0.35^aA^	4400.00
LysoPE	WS	0.03 ± 0.01^bC^	0.04 ± 0.01^bC^	0.04 ± 0.01^bB^	0.07 ± 0.02^aC^	133.33
	*PLDδ-def*	0.04 ± 0.01^aC^	0.04 ± 0.01^aBC^	0.05 ± 0.01^aB^	0.06 ± 0.02^aC^	50.00
	Col	0.06 ± 0.01^aB^	0.06 ± 0.00^aAB^	0.06 ± 0.02^aAB^	0.13 ± 0.05^bB^	116.67
	*PLDα1-def*	0.08 ± 0.01^aA^	0.07 ± 0.01^aA^	0.07 ± 0.02^aA^	0.23 ± 0.03^bA^	187.50
LysoPG	WS	0.34 ± 0.08^aA^	0.27 ± 0.09^aA^	0.01 ± 0.01^bA^	0.01 ± 0.00^bB^	−97.06
	*PLDδ-def*	0.18 ± 0.08^aB^	0.22 ± 0.09^aA^	0.01 ± 0.01^bA^	0.01 ± 0.01^bB^	−94.44
	Col	0.15 ± 0.12^bB^	0.28 ± 0.08^aA^	0.02 ± 0.01^cA^	0.02 ± 0.02^cB^	−86.67
	*PLDα1-def*	0.07 ± 0.03^bB^	0.21 ± 0.07^aA^	0.02 ± 0.01^cA^	0.05 ± 0.02^bcA^	−28.57

The RC is the percentage value between the 1100 Gy treated plants over the Control (0 Gy). Different lowercase letters marked in the same row represent significantly different among different γ-ray treatments. Different uppercase letters marked in the same column represent significantly different among different genotypes, (*p* < 0.05). Data are means ± standard deviation (*n* = 4 or 5)

### Levels of unsaturation and ACL of membrane glycerolipid change after γ-ray treatments

The DBI and ACL of glycerolipids are the two main determinants of the integrity and fluidity of membranes. The DBI of most phospholipids remained unchanged during treatment, with a minor decrease in PI (Table 4). Treatment with 1100 Gy caused an increase in the DBI of DGDG in the four genotypes of *Arabidopsis*. However, the levels of other chloroplastidic lipids, including MGDG and DGDG, decreased after treatment. The DBI of total lipids decreased significantly after γ-ray treatments in the four genotypes (Table 4). These results may indicate that glycerolipids with more double bonds degraded more rapidly during γ-ray-induced membrane injury, or that γ-rays may have oxidative effects on glycerolipids.

**Table 4:** Double-bond index of each lipid class after different doses of γ-ray treatment in WS, *PLDδ-deficient* (def), Col and *PLDα1-def* plants

Lipid species	Genotype	Dose of gamma irradiation (Gy)			
		0	46	210	1100
		Double-bond index			
DGDG	WS	5.21 ± 0.02^bA^	5.19 ± 0.08^bA^	5.15 ± 0.04^bA^	5.37 ± 0.03^aAB^
	*PLDδ-def*	5.20 ± 0.02^bA^	5.18 ± 0.04^bA^	5.13 ± 0.06^bAB^	5.34 ± 0.07^aB^
	Col	5.19 ± 0.08^bA^	5.13 ± 0.04^bcA^	5.08 ± 0.02^cB^	5.42 ± 0.07^aA^
	*PLDα1-def*	5.19 ± 0.03^bA^	5.18 ± 0.08^bA^	5.18 ± 0.02^bA^	5.39 ± 0.05^aAB^
MGDG	WS	5.92 ± 0.01^aAB^	5.91 ± 0.02^aA^	5.87 ± 0.04^bA^	5.84 ± 0.02^bAB^
	*PLDδ-def*	5.93 ± 0.01^aA^	5.93 ± 0.02^aA^	5.87 ± 0.02^bA^	5.87 ± 0.02^bA^
	Col	5.90 ± 0.03^aBC^	5.84 ± 0.05^abB^	5.85 ± 0.03^abA^	5.79 ± 0.08^bBC^
	*PLDα1-def*	5.89 ± 0.01^aC^	5.87 ± 0.02^abB^	5.84 ± 0.02^bA^	5.77 ± 0.05^cC^
PG	WS	3.20 ± 0.05^aA^	3.15 ± 0.05^aAB^	2.74 ± 0.12^bAB^	2.54 **±** 0.04^cAB^
	*PLDδ-def*	3.19 ± 0.04^aA^	3.19 ± 0.10^aA^	2.80 ± 0.12^bA^	2.59 ± 0.08^cAB^
	Col	3.14 ± 0.11^aA^	3.04 ± 0.09^aBC^	2.67 ± 0.08^bBC^	2.61 ± 0.07^bA^
	*PLDα1-def*	3.10 ± 0.12^aA^	2.98 ± 0.10^aC^	2.59 ± 0.02^bC^	2.51 ± 0.09^bB^
PA	WS	3.11 ± 0.11^aA^	3.24 ± 0.07^aAB^	3.05 ± 0.22^a^	2.61 ± 0.31^bB^
	*PLDδ-def*	2.92 ± 0.22^abA^	2.91 ± 0.25^abB^	2.77 ± 0.30^b^	3.19 ± 0.34^aA^
	Col	3.14 ± 0.28^aA^	3.30 ± 0.21^aAB^	3.13 ± 0.35^a^	3.34 ± 0.63^aA^
	*PLDα1-def*	3.23 ± 0.27^aA^	3.56 ± 0.60^aA^	3.18 ± 0.45^a^	3.29 ± 0.16^aA^
PC	WS	3.77 ± 0.05^abAB^	3.74 ± 0.02^abA^	3.70 ± 0.10^bA^	3.80 ± 0.02^aA^
	*PLDδ-def*	3.79 ± 0.10^aA^	3.82 ± 0.08^aA^	3.70 ± 0.02^bA^	3.77 ± 0.04^abA^
	Col	3.59 ± 0.17^abC^	3.54 ± 0.11^abB^	3.47 ± 0.16^bB^	3.71 ± 0.17^aA^
	*PLDα1-def*	3.61 ± 0.17^abBC^	3.51 ± 0.16^abB^	3.68 ± 0.04^aA^	3.45 ± 0.12^bB^
PE	WS	3.37 ± 0.02^bA^	3.42 ± 0.06^aA^	3.38 ± 0.04^abA^	3.31 ± 0.02^cAB^
	*PLDδ-def*	3.27 ± 0.04^bB^	3.29 ± 0.04^bB^	3.35 ± 0.04^aAB^	3.29 ± 0.02^bAB^
	Col	3.29 ± 0.02^aB^	3.28 ± 0.03^aB^	3.32 ± 0.02^aB^	3.34 ± 0.08^aA^
	*PLDα1-def*	3.27 ± 0.01^bcB^	3.30 ± 0.03^bB^	3.37 ± 0.03^aA^	3.25 ± 0.06^cB^
PI	WS	2.75 ± 0.02^aA^	2.76 ± 0.03^aA^	2.59 ± 0.03^bA^	2.59 ± 0.01^bB^
	*PLDδ-def*	2.68 ± 0.02^aB^	2.71 ± 0.02^aB^	2.61 ± 0.04^bA^	2.59 ± 0.03^bB^
	Col	2.69 ± 0.04^aB^	2.66 ± 0.03^abC^	2.54 ± 0.03^cB^	2.63 ± 0.02^bA^
	*PLDα1-def*	2.73 ± 0.04^aA^	2.69 ± 0.05^aBC^	2.58 ± 0.02^cA^	2.64 ± 0.04^bA^
PS	WS	2.66 ± 0.05^aA^	2.68 ± 0.01^aA^	2.67 ± 0.09^aA^	2.69 ± 0.07^aAB^
	*PLDδ-def*	2.67 ± 0.09^abA^	2.68 ± 0.06^aA^	2.58 ± 0.07^bA^	2.70 ± 0.05^aA^
	Col	2.66 ± 0.06^aA^	2.58 ± 0.08^abB^	2.48 ± 0.08^bB^	2.57 ± 0.12^abAB^
	*PLDα1-def*	2.66 ± 0.09^aA^	2.64 ± 0.03^aAB^	2.66 ± 0.05^aA^	2.55 ± 0.17^aB^
Total	WS	5.40 ± 0.02^aA^	5.36 ± 0.03^aA^	5.11 ± 0.15^bA^	4.82 ± 0.07^cA^
	*PLDδ-def*	5.38 ± 0.02^aA^	5.37 ± 0.06^aA^	5.12 ± 0.11^bA^	4.90 ± 0.14^cA^
	Col	5.33 ± 0.06^aAB^	5.18 ± 0.23^abA^	4.99 ± 0.26^bA^	4.50 ± 0.27^cB^
	*PLDα1-def*	5.29 ± 0.08^aB^	5.15 ± 0.24^abA^	4.97 ± 0.06^bA^	4.34 ± 0.23^cB^

Different lowercase letters marked in the same row represent significantly different among different γ-ray treatments. Different uppercase letters marked in the same column represent significantly different among different genotypes, (*p* < 0.05). Data are means ± standard deviation (*n* = 4 or 5)

Changes in ACL during γ-ray treatment were similar among the four genotypes, except for PI, which did not change in the WS ecotype, but exhibited a small decrease in the Col ecotype (Table 5). The ACL of the two galactolipids, PS, and the total lipids, increased during γ-ray-induced membrane injury, remained unchanged in PA, PC and PE, and exhibited a small decrease in PG (Table 5). The greatest change in ACL occurred for PS, which increased by more than one carbon in the WS and Col ecotypes (Table 5). The small increase in the ACL of total lipids after 1100-Gy treatment may have been due to the increase in the ACL of the most abundant lipids, MGDG and DGDG (Table 5). These results suggest that the rates of degradation of glycerolipids with acyl chains of different lengths may be similar.

**Table 5:** Acyl chain length of each lipid class after different doses of γ-ray treatment in WS, *PLDδ-deficient* (def), Col and *PLDα1-def* plants

Lipid species	Genotype	Dose of gamma irradiation (Gy)			
		0	46	210	1100
		Acyl chain length			
DGDG	WS	35.46 ± 0.02^bA^	35.44 ± 0.04^bcA^	35.41 ± 0.02^cB^	35.61 ± 0.02^aA^
	*PLDδ-def*	35.42 ± 0.01^bAB^	35.40 ± 0.03^bA^	35.40 ± 0.05^bB^	35.54 ± 0.05^aB^
	Col	35.42 ± 0.06^bAB^	35.39 ± 0.03^bA^	35.41 ± 0.02^bB^	35.64 ± 0.04^aA^
	*PLDα1-def*	35.40 ± 0.03^cB^	35.41 ± 0.06^cA^	35.47 ± 0.02^bA^	35.62 ± 0.03^aA^
MGDG	WS	34.32 ± 0.04^bA^	34.35 ± 0.06^bA^	34.36 ± 0.09^bAB^	34.50 ± 0.07^aAB^
	*PLDδ-def*	34.25 ± 0.02^cB^	34.26 ± 0.04^bcA^	34.34 ± 0.08^abB^	34.42 ± 0.08^aB^
	Col	34.25 ± 0.06^cB^	34.36 ± 0.18^bcA^	34.45 ± 0.15^abAB^	34.55 ± 0.12^aA^
	*PLDα1-def*	34.24 ± 0.04^cB^	34.39 ± 0.20b^cA^	34.48 ± 0.06^abA^	34.56 ± 0.09^aA^
PG	WS	33.86 ± 0.01^aB^	33.85 ± 0.02^aB^	33.88 ± 0.03^aA^	33.78 ± 0.03^bA^
	*PLDδ-def*	33.90 ± 0.02^aA^	33.89 ± 0.03^aA^	33.88 ± 0.03^aA^	33.82 ± 0.03^bA^
	Col	33.91 ± 0.02^aA^	33.91 ± 0.03^aA^	33.86 ± 0.02^bAB^	33.78 ± 0.03^cA^
	*PLDα1-def*	33.93 ± 0.03^aA^	33.90 ± 0.03^aA^	33.83 ± 0.02^bB^	33.66 ± 0.06^cB^
PA	WS	34.44 ± 0.11^abAB^	34.46 ± 0.17^abAB^	34.61 ± 0.17^aAB^	34.29 ± 0.26^bC^
	*PLDδ-def*	34.31 ± 0.20^aB^	34.32 ± 0.16^aB^	34.29 ± 0.22^aB^	34.52 ± 0.16^aBC^
	Col	34.59 ± 0.17^aA^	34.67 ± 0.26^aB^	34.68 ± 0.29^aAB^	34.79 ± 0.23^aA^
	*PLDα1-def*	34.66 ± 0.30^aA^	34.94 ± 0.64^aA^	34.83 ± 0.54^aA^	34.63 ± 0.06^aAB^
PC	WS	35.05 ± 0.05^bA^	35.01 ± 0.03^bA^	35.19 ± 0.04^aA^	35.14 ± 0.04^aA^
	*PLDδ-def*	35.18 ± 0.08^abA^	35.13 ± 0.04^bA^	35.25 ± 0.04^aAB^	35.16 ± 0.06^bA^
	Col	35.10 ± 0.17^aA^	35.10 ± 0.12^aA^	35.02 ± 0.17^aC^	35.05 ± 0.07^aA^
	*PLDα1-def*	35.08 ± 0.17^abA^	35.00 ± 0.22^abA^	35.11 ± 0.03^aBC^	34.89 ± 0.15^bB^
PE	WS	35.12 ± 0.05^bB^	35.11 ± 0.02^bB^	35.32 ± 0.05^aA^	35.17 ± 0.05^bA^
	*PLDδ-def*	35.21 ± 0.08^abA^	35.17 ± 0.03^bA^	35.29 ± 0.03^aA^	35.20 ± 0.09^bA^
	Col	35.17 ± 0.04^bAB^	35.18 ± 0.02^bA^	35.24 ± 0.02^aB^	35.17 ± 0.04^bA^
	*PLDα1-def*	35.19 ± 0.04^aA^	35.20 ± 0.05^aA^	35.23 ± 0.03^aB^	35.19 ± 0.07^aA^
PI	WS	34.13 ± 0.01^aC^	34.12 ± 0.02^abB^	34.11 ± 0.02^bAB^	34.11 ± 0.01^abA^
	*PLDδ-def*	34.15 ± 0.02^aBC^	34.13 ± 0.02^aB^	34.14 ± 0.06^aA^	34.11 ± 0.01^aA^
	Col	34.18 ± 0.04^aAB^	34.21 ± 0.05^aA^	34.09 ± 0.03^bB^	34.08 ± 0.02^bB^
	*PLDα1-def*	34.20 ± 0.01^aA^	34.18 ± 0.04^aA^	34.11 ± 0.01^bAB^	34.07 ± 0.04^cB^
PS	WS	40.47 ± 0.17^bA^	40.79 ± 0.12^aA^	40.97 ± 0.18^aA^	40.83 ± 0.25^aB^
	*PLDδ-def*	40.31 ± 0.20^cA^	40.55 ± 0.15^bA^	40.88 ± 0.12^aA^	41.00 ± 0.20^aAB^
	Col	39.62 ± 0.47^bB^	39.98 ± 0.40^bB^	40.72 ± 0.37^aA^	41.07 ± 0.35^aAB^
	*PLDα1-def*	39.78 ± 0.55^bB^	39.64 ± 0.49^bB^	40.72 ± 0.39^aA^	41.33 ± 0.48^aA^
Total	WS	34.58 ± 0.04^bA^	34.61 ± 0.05^bA^	34.65 ± 0.09^bAB^	34.82 ± 0.05^aAB^
	*PLDδ-def*	34.51 ± 0.02^cAB^	34.52 ± 0.07^cA^	34.63 ± 0.09^bB^	34.78 ± 0.07^aB^
	Col	34.48 ± 0.09^cB^	34.57 ± 0.13^bcA^	34.68 ± 0.10^bAB^	34.84 ± 0.03^aAB^
	*PLDα1-def*	34.48 ± 0.06^cB^	34.59 ± 0.16^cA^	34.74 ± 0.03^bA^	34.88 ± 0.07^aA^

Different lowercase letters marked in the same row represent significantly different among different γ-ray treatments. Different uppercase letters marked in the same column represent significantly different among different genotypes, (p < 0.05). Data are means ± standard deviation (n = 4 or 5)

## Discussion

γ-rays are high-energy ion irradiation that damage cells in a random manner [7]. Membranes are among the most important components of plant cells and are sensitive to environmental stress. Various abiotic stresses, such as freezing, phytotoxins, and pulse electric fields, can change the permeability and induce the breakdown of membranes, and the ion leakage of these plant tissues after treatment was more than 90% [22, 33, 34]. The effects of γ-rays on *Arabidopsis* growth were also shown to be dose-dependent, 2000 Gy γ-ray could inhibit growth and thereafter death of *Arabidopsis* [30], In our experiment, we treated *Arabidopsis* with γ-rays at very high doses of 1918 and 2885 Gy, which could cause growth inhibition in *Arabidopsis*. However, the ion leakage of leaves treated with these two doses was less than 10%, and the differences of ion leakage between wild-type and mutant plants were not statistically significant. Through their production of free radicals and ROS, γ-rays indirectly compromise the physiological and biochemical properties of cells [10–13], and induce deterioration of microsomal membranes [29]. In this study, we found that γ-rays caused increasement of ROS in *Arabidopsis*, and this accumulation of ROS may further induce the injury of membranes. The findings indicate that, during the process of γ-ray-induced plant death, the γ-rays may influence the membranes indirectly.

PLDδ and PLDα1 have different biochemical properties, subcellular associations, and gene expression patterns [35], and they play different roles in plant responses to freezing; in previous studies of *Arabidopsis*, the suppression of PLDδ caused freezing sensitivity [22] while the suppression of PLDα1 caused freezing tolerance [31]. However, in plant hormone induced senescence, the suppression of PLDδ and PLDα1 can retard the senescence in *Arabidopsis* [19, 28]. It is reported that γ-rays can induce a senescence-like deterioration in membranes [29]. However, we did not find any difference between the wild-type and the PLDδ and PLDα1 mutants under γ-ray-induced membrane injury, which may indicate that PLDδ and PLDα1 do not participate in *Arabidopsis* response to γ-rays.

Galactolipids and PG are the main plastidic lipids in chloroplast membranes [36]. In some stresses, such as low temperature and water deficit, the degradation of plastidic lipids was found to be more severe than that of extraplastidic ones [27, 37, 38]. Similarly, in this study, during γ-ray-induced membrane injury in *Arabidopsis*, the degradation of chloroplastidic lipids occurred more rapidly than that of extraplastidic ones, and the degradation of the most abundant glycerolipids, galactolipids, was most severe. The degradation of plastidic lipids may damage the photosystem and hence lead to senescence in *Arabidopsis*. The first plant process to be impaired by most types of stress is photosynthesis [39], and it has been suggested that chloroplast membrane collapse induced by membrane lipid degradation is the main reason for this.

PG is different from other phospholipids in that 34:1 and 34:2 PG molecules belong to the extraplastidic lipids, while 34:4 PG is the main glycerolipid in chloroplast membranes [19]. It was previously reported that 34:4 PG was markedly degraded during hormone-induced senescence in *Arabidopsis* [40]. PG can be degraded to PA by PLD or lysoPG by PLA [32]. In this study, γ-rays induced a marked degradation of PG in *Arabidopsis* (Table 1), but the level of lysoPG also decreased (Table 3). There were no major differences in the decrease in PG among WS, Col and their PLD mutant (Table 1). These results may indicate that γ-ray-induced PG degradation was not mainly due to PLA, PLDδ and PLDα1, but instead due to other enzymes such as PLC or other PLDs.

PC and PE are the main phospholipids in plasma membranes and are very important for membrane integrity and fluidity [10, 18]. The suppression of PLDα1 promoted PC and PE degradation during γ-ray-induced membrane injury (Table 1), and the production of PA, lysoPC and lysoPE led to their pronounced accumulation (Table 3). In the mutant of PLDδ, the decrease in PC was also greater than in WS (Table 1), but the change in lysoPC was not significant, the content of PE increased. PLDα1, PLA1 and PLDζ1 are the main enzymes participating in the degradation of PC, and PLDδ is also the main enzyme involved in phospholipid degradation [32]. The levels of lysoPC and lysoPE increased, which demonstrates that PLA-induced phospholipid degradation may be the main pathway of PC and PE degradation. The findings suggest that γ-ray-induced PC degradation was caused mainly by PLDζ1 and PLA1, which caused PA and lysoPC accumulation.

PA is an important lipid that can act as a signalling or structural molecule under stress [38]. Its level can increase several fold, or sometimes more than tenfold, in plants in response to stress, and decline to the control level during recovery [27]. The molecular shape of PA is cone-like, and its pronounced accumulation can cause membrane leakage [27]. The level of PA can even increase 100 times after freezing treatment and be maintained at that level, which may be one of the most important factors behind plant death [38]. However, PLD-induced phospholipid degradation and PA accumulation-induced death may not be the reasons for the γ-ray-induced membrane injury in *Arabidopsis*. During γ-ray-induced membrane injury, the changes in PA in the two wild-type *Arabidopsis* and the PLDδ mutant were less than 90%, while PA increased more than 20 times in the PLDα1 mutant (Table 1). The major increase in PA in the PLDα1 mutant may suggest that mechanisms of PA production other than PLDα1-induced phospholipid degradation compensate for PLDα1 during γ-ray-induced membrane injury when PLDα1 is inhibited. In addition, this compensation effect involved far more than just PLDα1-induced accumulation of PA. During γ-ray induced membrane injury, PLDδ and PLDα1 may not participate in the processes of lipid degradation and PA did not accumulate, which is different from hormone or low temperature stresses [19, 28, 38].

DBI and ACL of lipids are two of the most important determinants of membrane fluidity, which are also closely correlated to the environment stresses and growth of plants [15, 18]. An increase in DBI and a decrease in ACL may make the membranes more fluid, and vice versa. During γ-ray-induced membrane injury, the DBI of the total lipids decreased and the ACL increased, in all four genotypes of *Arabidopsis*. In addition, the most pronounced changes occurred in chloroplast membranes, such as in galactolipids and PG. Furthermore, the ACL of PS increased, which may be correlated to the lifespan of plants, as we reported previously [15]. This suggests that γ-ray treatment could make membranes more rigid, which may have further harmful effects on their biochemical reactions, and thereafter induce the membrane injury.

## Conclusions

The present study investigates metabolism of glycerolipidome during γ-rays induced membrane injury, and the possible roles of PLDδ and PLDα1 in this process. Although γ-rays are high-energy ionic radiation that can cause random damage to cells, a high dose of γ-rays indirectly damages membranes. Mutations of PLDδ and PLDα1 had little effect on plant responses to γ-rays. Most γ-ray-induced glycerolipid degradation occurred in chloroplastidic lipids rather than extraplastidic ones. During γ-ray-induced membrane injury, the degradation of PC and PE may occur mainly through PLDζ1 and PLA1; however, the degradation of PG was not through PLA, PLDδ and PLDα1, but rather through PLC or other PLDs. γ-ray-induced lipid degradation may be moderate, which does not cause any great accumulation of PA. γ-ray-induced oxidative stresses may cause a decrease in DBI and an increase in ACL, which can make the membrane more rigid and in turn may cause injury of membrane.

## Materials and methods

### Plant materials and treatments

*PLDδ-knockout* mutant (*PLDδ-def*) had previously been isolated from *Arabidopsis* [Wassilewskija (WS)]. *PLDα1-deficient* mutant (*PLDα1-def*) was generated by antisense suppression from *Arabidopsis* [Columbia ecotype (Col)]. The mutants were confirmed by the absence of the transcript, protein, and activity of PLDα1 or PLDδ [28, 41]. The conditions of our growth chamber are 22/18 °C (day/night), light quantum of 120 μmol·m^−2^·s^−1^, 60% humidity, and 12-h photoperiod. The γ-irradiation treatment was performed by Yunnan Hua Yuan Nuclear Radiation Technology Co., Ltd. Twenty-day-old plants were treated with γ-rays at 46, 210, 1100, 1918 or, 2885 Gy with a ^60^Co source (25 Gy min^−1^). Control plants were placed in the dark close to the irradiation platform. After treatment, irradiated and control seedlings were returned to the growth chamber for 25 days and then harvested for plant lipids.

### Measurement of ion leakage

Measurement of ion leakage was performed as described previously, with minor modifications [31]. Twenty-day-old plants were washed briefly with deionised water before gamma irradiation treatment to remove surface-adhered electrolytes. To measure ionic leakage, after irradiation two leaves of each treatment group were immersed in 5 ml of deionised water; the conductivity of the solution was then measured after gentle agitation at 23 °C for 3 h (*C*_1_). Total conductivity was determined after boiling the solution for 10 min and then cooling it to 23 °C (*C*_2_). Ion leakage was calculated as the percentage of the initial conductivity compared with the total conductivity [ion leakage (%) = *C*_1_/*C*_2_ × 100%].

### Intracellular reactive oxygen species (ROS) measurement

Intracellular ROS was measured using the ROS-sensitive dye 2′,7′-dichlorofluorescin diacetate (H_2_DCFDA) which purchased from Sigma (D6883). Leaves of 7-day-old *Arabidopsis* were treated by various doses of gamma irradiation, and stained with 5 μg ml^−1^ H_2_DCFDA immediately after treatment, then rinsed three times with MS liquid medium. After staining and rinsing, the leaves were observed under a confocal laser scanning microscope (FV-1000; Olympus, Japan). Images were processed with Adobe Photoshop CS.

### Test of lipid peroxidation

The level of lipid peroxidation was estimated by content of malondialdehyde (MDA) according to Duan et al. [42]. After gamma irradiation treatment, 0.5 g of fresh leaves was ground into powder and homogenized in 4 ml of prechilled 10% (*w*/*v*) trichloroacetic acid (TCA) reagent, then centrifuged at 12,000 g for 10 min. After that, 2 ml of supernatant was added to 2 ml 0.6% (w/v) thiobarbituric acid (TBA) and incubated in boiling water for 30 min. After cooling and centrifugation, the absorption of the supernantant was determined at 450, 532 and 600 nm. The content of MDA was calculated using the following formula: C (nmom ml-1) = 6.45 × (A_532_-A_600_)-0.56 × A_540_.

### Lipidomic analysis

Lipidomic analysis was performed according to previous studies, with minor modifications [31, 43] (Kansas Lipidomics Research Center, http://www.k-state.edu/lipid/lipidomics). Fresh leaves of *Arabidopsis* were put into 3 ml of pre-heated (75 °C) isopropanol (0.01% butylated hydroxytoluene) to inhibit lipolytic activity. Then the leaves were extracted with chloroform/methanol (2:1) three times (each time for 12 h). After extraction, the leaves were dried at 105 °C overnight and weighed for dry mass. The lipid samples were dried under N_2_ gas at room temperature for analysis. Data processing was performed as described previously [31, 43]. There are five replicates for each treatment.

### Statistical procedures

Data from discordant samples were removed according to Q-test [42]. SPSS 13.0 was used during the analysis of one-way ANOVA and principal component analysis (PCA). Fisher’s least significant difference method was used for analysis of significance among data. Data were experienced hierarchal clustering analysis (HCA) through Cluster 3.0 and Java TreeView. The double-bond index (DBI) and acyl chain length (ACL) were calculated as follows: DBI = (∑[*N* × mol% lipid])/100, ACL = (∑[n × mol% lipid])/100, where N and n is the number of double bonds and acyl carbons in each lipid molecule respectively.

## Additional files

Additional file 1: **Figure S1.** Growth condition of *Arabidopsis* after different doses of γ-rays treatment for 10 days. WS, Wassilewskija ecotype; *PLDδ-def*, *PLDδ-knockout* mutant with WS background; Col, Columbia ecotype; *PLDα1-def*, *PLDα1-deficient* mutant with Col background (JPEG 915 kb)

Additional file 2: **Figure S2.** The subcellular distribution of ROS after different doses of γ-rays treatment. *Arabidopsis* seedlings were stained with 5 μg ml^−1^ H_2_DCFDA, and observed under a confocal laser scanning microscope. Bars = 50 μm. AF, autofluorescence; WS, Wassilewskija ecotype; *PLDδ-def*, *PLDδ-knockout* mutant with WS background; Col, Columbia ecotype; *PLDα1-def*, *e* mutant with Col background (JPEG 3420 kb)

Additional file 3: **Figure S3.** Levels of MDA after various doses of gamma irradiation treatment. Blank bars represent control, black bars represent 2060 Gy gamma ray treated plants, and light grey bars represent 2786 Gy gamma ray treated plants (TIFF 769 kb)

## Abbreviations

ACL: Acyl chain length
DBI: Double bond index
DGDG: Digalactosyldiacylglycerol
lysoPC: lysophosphatidylcholine
lysoPE: lysophosphatidylethanolamine
lysoPG: lyso phosphatidylglycerol
MDGD: Monogalactosyldiacylglycerol
PA: Phosphatidic acid
PC: Phosphatidylcholine
PE: Phosphatidylethanolamine
PG: Phosphatidylglycerol
PI: Phosphatidylinositol
PLD: Phospholipase D
PS: Phosphatidylserine
ROS: Reactive oxygen species
