# Supplementary figures and images for: Profiling membrane glycerolipids during γ-ray-induced membrane injury
Source: BMC Plant Biol. 2017 Nov 15;17:203. doi: 10.1186/s12870-017-1153-9 (PMC5688707; doi:10.1186/s12870-017-1153-9)

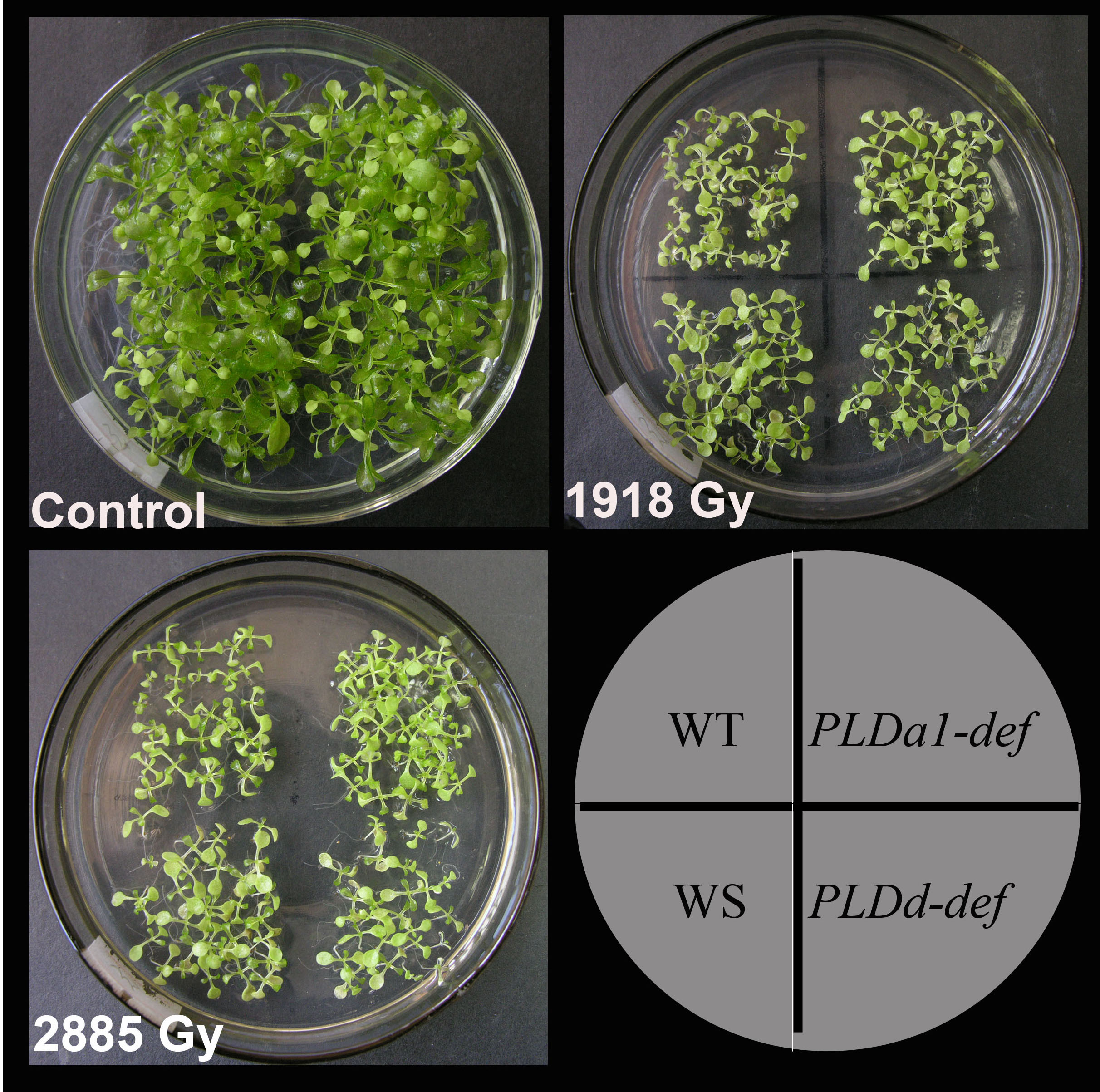

Supplement: Supplementary file 1 — Figure S1. Growth condition of Arabidopsis after different doses of γ-rays treatment for 10 days. WS, Wassilewskija ecotype; PLDδ-def, PLDδ-knockout mutant with WS background; Col, Columbia ecotype; PLDα1-def, PLDα1-deficient mutant with Col background (JPEG 915 kb) [file 12870_2017_1153_MOESM1_ESM.jpg]

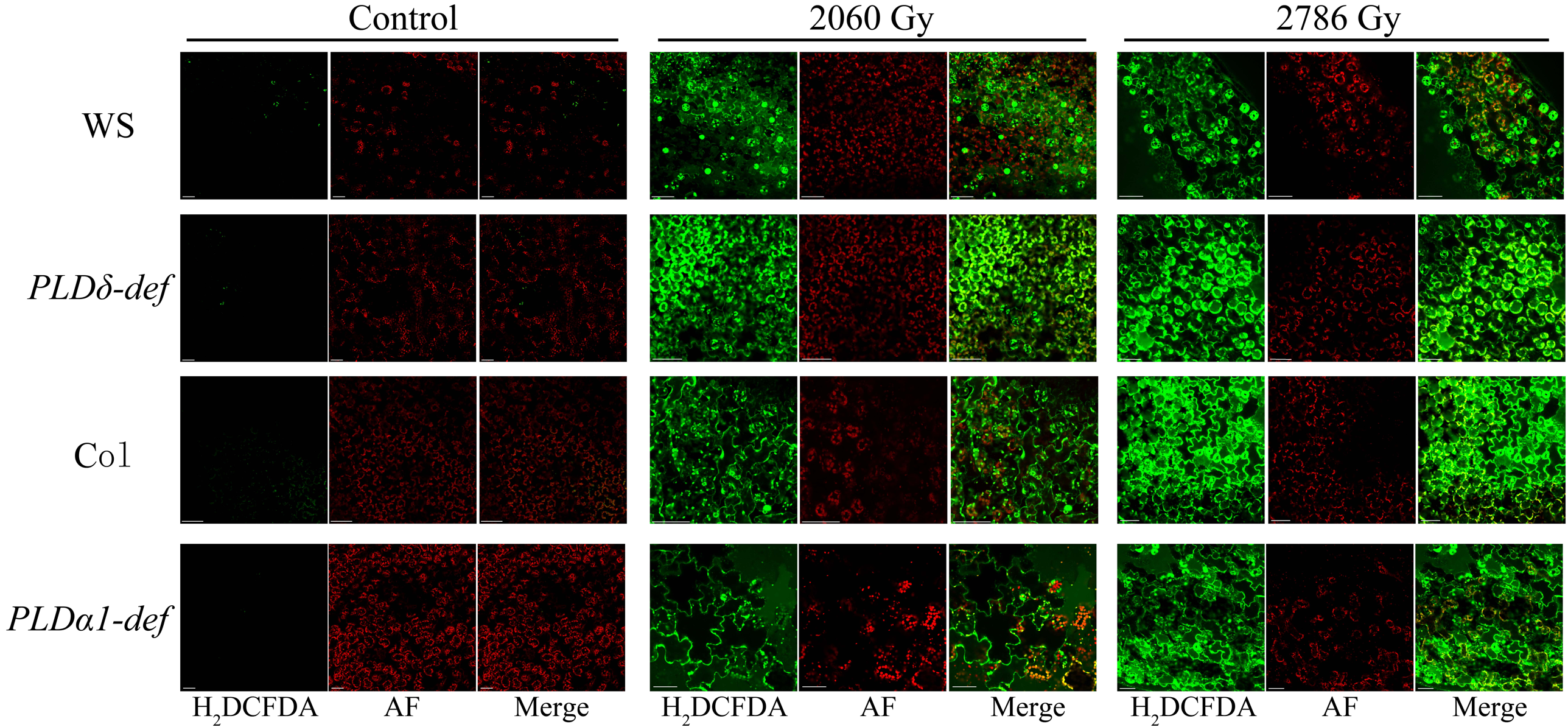

Supplement: Supplementary file 2 — Figure S2. The subcellular distribution of ROS after different doses of γ-rays treatment. Arabidopsis seedlings were stained with 5 μg ml−1 H2DCFDA, and observed under a confocal laser scanning microscope. Bars = 50 μm. AF, autofluorescence; WS, Wassilewskija ecotype; PLDδ-def, PLDδ-knockout mutant with WS background; Col, Columbia ecotype; PLDα1-def, e mutant with Col background (JPEG 3420 kb) [file 12870_2017_1153_MOESM2_ESM.jpg]

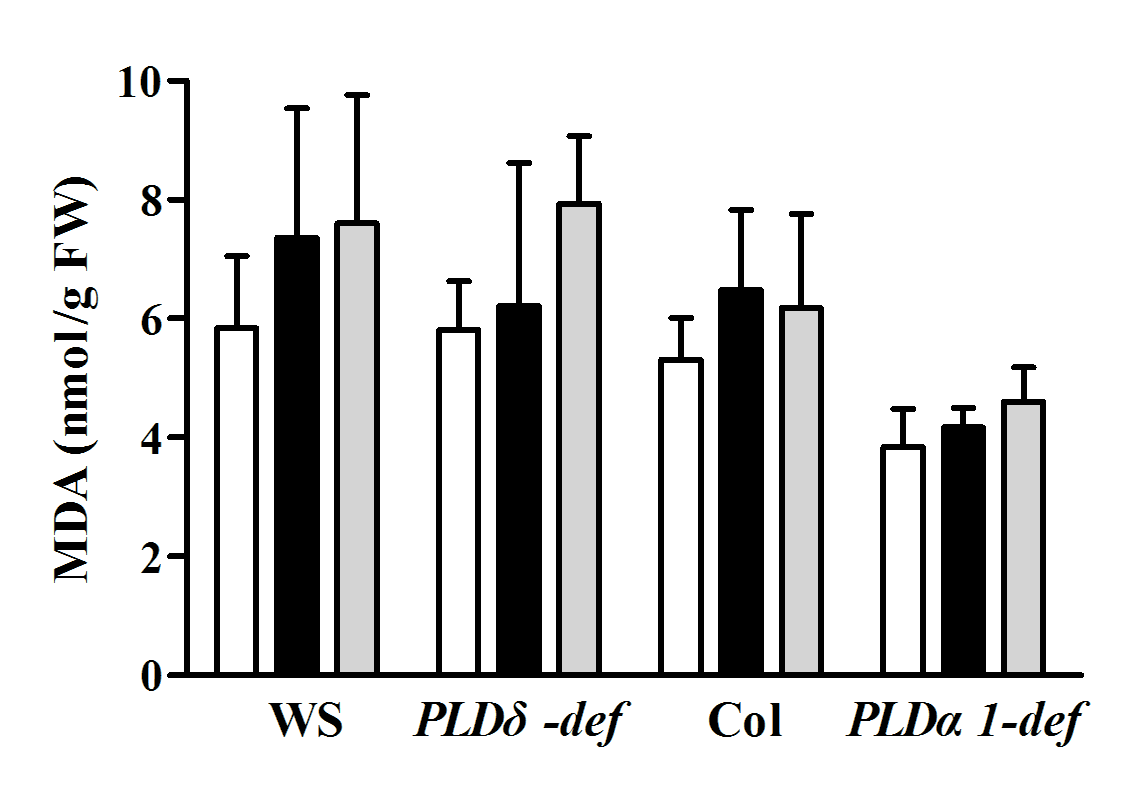

Supplement: Supplementary file 3 — Figure S3. Levels of MDA after various doses of gamma irradiation treatment. Blank bars represent control, black bars represent 2060 Gy gamma ray treated plants, and light grey bars represent 2786 Gy gamma ray treated plants (TIFF 769 kb) [file 12870_2017_1153_MOESM3_ESM.tif]
